# Cryoprotective Activity of Different Characterized Fractions Isolated from Enzymatic Hydrolysates of Croceine Croaker (*Pseudosciaena crocea*)

**DOI:** 10.3390/foods13121946

**Published:** 2024-06-20

**Authors:** Zhe Xu, ShengAo Cao, Na Cui, Rui Zhang, Shuang Zhao, Lijuan Zhang, Shuang Guan, Yikun Xu, Xu Yan, Zhixuan Zhu, Zhijian Tan, Tingting Li

**Affiliations:** 1 Key Laboratory of Biotechnology and Bioresources Utilization, College of Life Sciences, Dalian Minzu University, Ministry of Education, Dalian 116600, China; dlpuxz@163.com (Z.X.); csa991102@163.com (S.C.); 18842647008@163.com (S.Z.); 20231575@dlnu.edu.cn (L.Z.); 19818957899@163.com (S.G.); 18078653937@163.com (Y.X.); yx13752409672@163.com (X.Y.); zzx_0727@126.com (Z.Z.); 2 Institute of Bast Fiber Crops & Center of Southern Economic Crops, Chinese Academy of Agricultural Sciences, Changsha 410205, China; 3 Department of Food and Chemical Engineering, Liuzhou Institute of Technology, Liuzhou 545616, China; 15678068121@163.com; 4 National Engineering Research Center of Seafood, Collaborative Innovation Center of Seafood Deep Processing, School of Food Science and Technology, Dalian Polytechnic University, Dalian 116034, China; zhangr9965@163.com

**Keywords:** cryoprotective, isolation, anti-freezing, trypsin hydrolysates, myofibrillar protein

## Abstract

In this study, ultrafiltration fractions (<3 k Da, LMH; >3 k Da, HMH) and solid-phase extraction fractions (hydrophilic hydrolysate, HIH; hydrophobic hydrolysate, HOH) from trypsin hydrolysate purified from croceine croaker (*Pseudosciaena crocea*) isolate were obtained to investigate the cryoprotective effects of the different fractions, achieved by means of maceration of turbot fish meat after three freeze-thaw cycles. Alterations in the texture, color, moisture loss, myofibrillar protein oxidation stability and conformation, and microstructure of the fish were analyzed after freezing and thawing. The results demonstrate that HIH maximized the retention of fish texture, reduced moisture loss, minimized the oxidation and aggregation of myofibrillar proteins, and stabilized the secondary and tertiary structures of myofibrillar proteins compared to the control group. In conclusion, the HIH component in the trypsin hydrolysates of croceine croaker significantly contributes to minimizing freeze damage in fish meat and acts as an anti-freezing agent with high industrial application potential.

## 1. Introduction

Marine fish, recognized for their tender texture and high nutritional value, are commercially significant species in the aquaculture industry [1]. However, due to their high moisture and protein contents, marine fish are particularly prone to spoilage. To solve this problem, cryopreservation has emerged as the leading method for effectively reducing metabolic rates in aquatic products and preserving their quality [2,3]. Despite its effectiveness, cryopreservation faces challenges related to temperature fluctuations during storage and transport, which often lead to moisture crystallization during freeze-thaw cycles, potentially compromising product quality. Studies have shown that ice crystal formation can detrimentally affect the quality of aquatic products by causing cellular damage and protein denaturation [4]. Thus, the implementation of an efficient cryoprotectant is critical [5]. The traditional cryoprotectant, consisting of a 4% mixture of sorbitol and sucrose, is widely used in commercial aquatic products; however, its unnecessary caloric value and pronounced sweetness have driven consumer demand for non-sugar-based alternatives [6].

Antifreeze peptides have demonstrated significant potential in inhibiting ice crystal growth through lowering the freezing point of water [7]. Some researchers have found that certain protein hydrolysates, similarly to cryoprotectants, have the ability to interact with specific components to enhance their structural integrity and stability [8]; for example, the membrane-separated fraction of silver carp hydrolysate can stabilize the structure of myosin through hydrogen bonding, electrostatic interactions, and hydrophobic interactions and, as such, is expected to serve as a novel cryoprotective agent [9]. Wu et al. isolated a collagen peptide from silkworm cocoon, which showed good performance in inhibiting ice crystal growth and protecting probiotics from frostbite [10]. It has been reported that antifreeze peptides generally have specific amino acid sequence lengths and molecular weights less than 2 kDa [11,12]. Our prior study reported that a tryptic hydrolysate (TH) of croceine croaker significantly enhanced the physicochemical attributes of fillets, including their water-holding capacity, texture, oxidative stability, and thermal stability after cold storage, presenting comparable antifreeze capabilities to traditional agents [13]. However, few studies have assessed the cryoprotective effects and mechanisms in fish after the isolation and purification of antifreeze peptides.

To investigate the industrial application potential, the presented research involved successively isolating distinct fractions through ultrafiltration and solid-phase extraction, focusing on identifying the antifreeze-active components present in the TH extract from croceine croaker. The evaluation of the antifreeze efficacy of these components involved studying the physical attributes of turbot muscle following three freeze-thaw cycles, along with the oxidative and structural stability of myofibrillar proteins. This research enhances our comprehension of antifreeze peptides derived from marine fish, and it lays the groundwork for their utilization in the food industry.

## 2. Experimental Materials and Methods

### 2.1. Materials and Reagents

Croceine croaker and turbot were obtained from Dalian RICH FOODS Co., Ltd. (Liaoning, China). Trypsin (bovine pancreas) was purchased from Yuan Ye Science & Technology Co., Ltd. (Shanghai, China), and bovine serum albumin (BSA) was purchased from Solarbio Science & Technology Co., Ltd. (Beijing, China). The remaining substances were all of analytical grade.

### 2.2. Preparation of Protein Hydrolysate from Croceine Croaker

The tissue muscle of croceine croaker was homogenized (IKAT18, IKA, GER) in a 1:3 (*w*/*v*) ratio with deionized water, followed by magnetic stirring for 4 h at 4 °C and centrifugation at 10,000× *g* (H1750R, Cence, CHN) for 10 min at 4 °C to separate the supernatant. The obtained supernatant was subsequently lyophilized for 12 h to yield a water-soluble protein extract. Trypsin was added with 5000 U/g protein in the water-soluble protein extract. The mixture was incubated in a water bath at 37 °C with a pH of 8.0 for 5 h to facilitate enzymatic hydrolysis. Subsequent enzyme inactivation was achieved by heating at 100 °C for 10 min, followed by pH adjustment to 7.0. The mixture was then centrifuged at 10,000× *g* for 10 min at 4 °C. The final supernatant was frozen and lyophilized in a freeze drier (Labconco, Kansas City, MO, USA) in order to obtain the tryptic hydrolysate (TH) of croceine croaker.

### 2.3. Pre-Separation of Enzymatic Hydrolysates of Croceine Croaker by Ultrafiltration (UF)

TP powder was dissolved in deionized water, then separated using a UF centrifugal filtration device (Amicon Ultra-4, Millipore, Burlington, MA, USA) equipped with a 3 kDa molecular weight cutoff membrane. The resulting fractions were classified based on molecular weight; fractions exceeding 3 kDa were designated as high-molecular-weight hydrolysates (HMH), whereas those below 3 kDa were designated as low-molecular-weight hydrolysates (LMH). Both fractions were then subjected to lyophilization in preparation for the assessment of their antifreeze activities.

### 2.4. Fractionation of Enzymatic Hydrolysates of Croceine Croaker by Solid-Phase Extraction (SPE)

The UF fraction with the best cryoprotection was further subjected to fractionation using a reversed-phase C18 solid-phase extraction column (Cleanert ODS solid, Agela, Wilmington, DE, USA). Prior to the extraction process, the adsorbent was activated using methanol and subsequently eluted with water. The UF fraction was then introduced into the column, and the initial effluent was collected as the hydrophilic hydrolysates (HIH). After elution with water and methanol, the hydrophobic hydrolysates (HOH) were collected. Assessment of antifreeze activity for both HIH and HOH fractions was conducted employing the methodology introduced in the following section.

### 2.5. Sample Pre-Treatment

Turbot samples were processed as described in our previous study [13,14]. Three single turbots were immediately stunned, slaughtered, and skinned, then preserved on ice for transportation to the laboratory within 1 h before the fish presented rigor mortis. During the experiment, each fish yielded four fillets, each of which weighed approximately 40 g. Each of the five separate groups of fillets was then individually combined with 2 mg/mL of cryoprotectant. The cryoprotectant treatments included the application of LMH, HMH, HIH, HOH, and TH solutions. Additionally, two more groups of fillets were taken to set up two control groups: the fresh fish (FF) group, which received no treatment, and a negative control group (Control), in which samples were mixed with an equivalent volume of water as used in the treatment solutions. The samples for treatment were submerged in their respective solutions for a duration of 4 h at 4 °C, after which they were subjected to three freeze-thaw cycles. A complete freeze-thaw cycle entailed freezing at −20 °C for a duration of 24 h, followed by thawing at 4 °C for 12 h, concluding once the core temperature reached the range of 0~4 °C.

### 2.6. Extraction of Turbot Myofibrillar Protein (MFP)

MFP was extracted from turbot fish fillets after each freeze-thaw cycle according to the method described by Cao et al. [15]. Post-thaw, the muscle tissue was sectioned into a fine particulate form and subsequently re-suspended in a 20 mmol/L phosphate buffer (pH 7.0) at a volumetric ratio of 1:4 (*w*:*v*). This suspension was subjected to homogenization at 4000 rpm, followed by centrifugation for 15 min (4500× *g*, 4 °C) to separate the precipitate. This centrifugation step was iteratively performed thrice, with the final cycle incorporating a modified phosphate buffer (20 mmol/L; containing 0.6 M NaCl, adjusted to pH 6.7) to enhance protein solubilization. The supernatant enriched with MFP was subsequently collected through filtration using sterile gauze and maintained at 4 °C for no more than 48 h. The protein content of MFP was determined according to a standard curve plotted with BSA content and determined using the biuret method [16].

### 2.7. Determination of Physical Properties

#### 2.7.1. Texture Properties

The fish fillet samples were sectioned into 1 cm^3^ cubic segments for texture analysis (TA-XT plus, Stable Micro Systems, Godalming, UK), and 10 measurements were performed for each sample. The parameter settings referred to the method provided by Cai et al. [17], and hardness, elastic, cohesion, adhesion, chewiness, and reversibility parameters were determined for all samples using a P/50 probe in 30% strain mode and a test speed of 1.0 mm/s throughout, with other settings set at default.

#### 2.7.2. Moisture Loss

The moisture loss after the thawing process was determined according to the literature [18]. In brief, the fish fillet samples were thawed until their core temperature reached 4 °C, then dried with filter paper. The fish fillet samples were weighed both before and after they were unfrozen, denoted as T_0_ and T_1_, respectively. The moisture loss after the thawing process was calculated according to Equation (1):

(1) $$\mathrm{Thawing}\;\mathrm{loss}\;(\%)=\frac{T_{0}-T_{1}}{T_{0}}\times 100\%$$

The moisture loss after the centrifugal process was determined according to the literature, with slight modifications [19]. The mass of fish fillets before and after centrifugation was weighed and denoted as T_2_ and T_3_, respectively. The moisture loss after the centrifugal process was calculated according to Equation (2):

(2) $$\mathrm{Centrifugal}\;\mathrm{loss}\;(\%)=\frac{T_{2}-T_{3}}{T_{2}}\times 100\%$$

The moisture loss after cooking was determined according to the literature, with slight modification [19]. Thawed fish fillets (T_4_) were packed in polyethylene bags and steamed at 85 °C for about 20 min before being removed, cooled to room temperature, and dried and weighed (T_5_). The cooking loss was calculated according to Equation (3):

(3) $$\mathrm{Cooking}\;\mathrm{loss}\;(\%)=\frac{T_{4}-T_{5}}{T_{4}}\times 100\%$$

#### 2.7.3. Color

The color of the frozen and thawed fish fillet samples was determined using a colorimeter (CR-400, Konica Minolta, Tokyo, Japan). All samples were measured six times in parallel to obtain the color parameters of L* (lightness value), a* (redness value), and b* (yellowness value).

#### 2.7.4. Electronic Nose (E-Nose)

Volatile odors of individual fish fillet samples were detected using an E-nose (PEN 3, Insent, Atsugi, Japan). Each group of fish fillet samples was ground and placed in a measuring bottle, then sealed with plastic wrap. The test needle was inserted through the plastic wrap during measurements. Each measurement lasted for a time period of 60 s, with a purge air of 60 s and an instrumental flow rate of 200 mL/min.

### 2.8. Determination of MFP Conformation

#### 2.8.1. Fluorescence Spectroscopy

The fluorescence spectrum of MFP was scanned using a fluorescence spectrometer (Fluorolog-3, Horiba, Jobin Yvon, Longjumeau, France), according to the method described by Cao et al. [15] with a minor modification. The concentration of the MFP sample was 0.1 mg/mL, and the fluorescence images were obtained at the excitation wavelength of 295 nm and the emission wavelength of 300~450 nm.

#### 2.8.2. UV Absorption Spectroscopy

The UV spectrum of the extracted MFP solution (0.5 mg/mL) was detected using a spectrophotometer (PerkinElmer, Salem, MA, USA). The wavelength range of 200~400 nm was scanned, with PBS solution used as a reference.

#### 2.8.3. Circular Dichroism (CD)

The secondary structure of the extracted MFP sample was determined using a circular dichroism spectrometer (Chirascan VX, Applied photophysics, Leatherhead, UK), according to previously reported methods with minor modifications [20]. The 0.2 mg/mL MFP samples were measured in the wavelength range of 200~250 nm. The PBS solution was used as a blank reference, and the secondary structure was analyzed.

### 2.9. Scanning Electron Microscope (SEM)

The microstructure of pre-treated samples was measured using an SEM (S-4800, Hitachi, Tokyo, Japan), according to the method described by Cai et al. [21]. Briefly, the samples were cut into 1 × 1 × 1 mm^3^ pieces and soaked in 2.5% glutaraldehyde (4 °C) for 24 h, followed by dehydration using a gradient of 30% to 100% alcohol 3 times (10 min each time). The samples were then lyophilized and sprayed with a layer of gold for SEM observation with 5 kV voltage and a magnification of 1000×.

### 2.10. Determination of Frozen Denaturation and Oxidation of MFP

#### 2.10.1. Particle Size and Zeta Potential

Particle size was determined according to the study protocol described by Cai et al. [22]. MFP was diluted to 2 mg/mL and filtered through a polyethersulfone aqueous filter membrane (0.45 μm). The particle size was measured using a laser nanoparticle sizer (Zetasizer Pro, Malvern, UK). Zeta potential testing was performed after switching modes of the same instrument, and the pre-treatment for the determination of sample zeta potential was the same as that for particle size determination, with water as the solvent.

#### 2.10.2. Surface Hydrophobicity

The So-ANS of MFP was determined using the 8-anilino-1-naphthalene sulfonate (ANS) fluorescent probe [23]. Briefly, 2 mL of MFP solution 0.2~1.0 mg/mL was mixed with 10 μL of 20 mmol/L phosphate buffer (8 mM ANS, 0.6 M NaCl, pH 6.7) for the measurement of fluorescence intensity at an excitation wavelength of 390 nm and emission wavelength of 470 nm using a fluorescence photometer (970 CRT, Shanghai Precision and Scientific Instrument Co., Ltd., Shanghai, China). The So-ANS is expressed according to the slope of fluorescence intensity against protein concentration.

#### 2.10.3. Dityrosine Content

The dimerized tyrosine content of MFP samples was determined using a fluorescence spectrophotometer, according to the literature [24]. The concentration of each group of MFP was adjusted to 1 mg/mL, then measured at an excitation wavelength of 325 nm and emission wavelength of 420 nm. The dimerized tyrosine levels are expressed as fluorescence values (AU).

#### 2.10.4. Ca^2+^-ATPase Activity

The MFP assay was performed using the ultra-trace Ca^2+^-ATPase test kit (A070-3, Jiancheng, China). The sample MFP (1 mg/mL) was tested according to the protocols in the instruction manual, and the absorbance (OD value) was measured at 636 nm using a UV spectrophotometer (UV-1600, Mapada, Shanghai, China). Ca^2+^-ATPase activity was calculated according to Formula (4):

(4) $${\mathrm{Ca}}^{2+}\text{-}\mathrm{ATPase}\;\mathrm{activity}\left(U/\mathrm{mgprot}\right)=\frac{A_{c}-A_{d}}{A_{b}-A_{k}}\times C_{s}\times 6^{*}\times 7.8^{**}÷C_{MFP}$$

where *A_c_*, *A_d_*, *A_b_*, and *A_k_* are the OD values of the assay, control, standard, and blank samples, respectively; *C_s_* is the phosphorus standard concentration (0.02 μmol/mL); 6* represents 1 h, as each actual operation took 10 min; 7.8** shows that the reaction system was diluted 7.8-fold; and *C_MFP_* is the MFP concentration (1 mg/mL).

#### 2.10.5. Protein Solubility

The solubility of MFP was assessed according to the literature [22]. In brief, 3 mg/mL of MFP solution was centrifuged for 15 min (4500× *g*, 4 °C) to obtain the supernatant. The protein solubility is calculated as the percentage of protein content of the supernatant divided by the solution protein content (3 mg/mL).

#### 2.10.6. Total Sulfhydryl Content

The MFP assay was performed using a micro total sulfhydryl test kit (A063-2-1, Jiancheng, China). The sample MFP (1 mg/mL) was tested according to the instruction manual, and the OD value was measured at 405 nm using an enzyme meter (Synergy H1, Biotek, Paramus, NJ, USA). Total sulfhydryl content was calculated according to Equation (5):

(5) $$\mathrm{Total}\;\mathrm{sulfhydryl}\;\mathrm{content}\left(\mu \mathrm{mol}/L\right)=\frac{A_{c}-A_{d}}{A_{b}-A_{k}}\times C_{s}÷C_{\mathrm{MFP}}$$

where *A_c_*, *A_d_*, *A_b_*, and *A_k_* are the OD values of the assay, control, standard, and blank samples, respectively; *C_s_* denotes the standard concentration (500 μmol/L); and *C_MFP_* is the MFP concentration (1 mg/mL).

#### 2.10.7. Carbonyl Content

The MFP assay was performed using a protein carbonyl content test kit (A087-1, Jiancheng, China). The sample MFP (1 mg/mL) was tested according to the instruction manual, and the OD value (370 nm) was obtained using a UV spectrophotometer (UV-1600, Mapada, China). Carbonyl content was calculated according to Formula (6):

(6) $$\mathrm{Carbonyl}\;\mathrm{content}\left(\mathrm{nmol}/\mathrm{mgprot}\right)=\frac{A_{c}-A_{d}}{22\times d\times C_{MFP}}\times 125\times 10^{5}$$

where *A_c_* and *A_d_* are the OD values of the measured and control samples, respectively; *d* is the colorimetric optical diameter (cm); and *C_MFP_* is the MFP concentration (1 mg/mL).

### 2.11. Statistical Analysis

Data were analyzed using one-way analysis of variance (ANOVA) with the SPSS 26 software (IBM Corp., Armonk, NY, USA), and significance was analyzed using the Waller-Duncan Multiple Extreme Variance Test (*p* < 0.05). Images were plotted using Origin 2021 (OriginLab Co., Northampton, MA, USA), and all experiments were performed three or more times.

## 3. Results and Discussion

### 3.1. Analysis of Texture Properties

During the repeated freezing and thawing process, the expansion of ice crystals causes the breakdown of cell membranes and organelles, as well as the destruction of protein structures, which eventually causes the texture of meat to become soft as its muscle structure is damaged [25,26]. As shown in Table 1, the hardness and cohesiveness of LMH were significantly higher than those of the control group (*p* < 0.05); moreover, the average hardness and cohesiveness values were also higher than those for the HMH and TH groups. After further isolation of LMH, the hardness of HIH was significantly higher than that of the treatment groups (*p* < 0.05), while the elasticity and cohesiveness did not change significantly when compared with the other treatment groups (LMH, HMH, HOH, and TH). The highest hardness value of HIH may be due to its minimal water loss after freeze-thaw treatment (Table 2). In addition, the results show that HIH reduced the damage of ice crystals to muscle cells and maintained the stability of the protein structure, thus increasing the hardness.

### 3.2. Analysis of Color

Freezing altered the surface color of the fish meat, with variations in L*, a*, and b* values across different samples post-cryopreservation, as detailed in Table 2. The LMH-treated samples exhibited significantly higher L* and a* values compared to the control, following the order of LMH > TH > HMH (*p* < 0.05), whereas their b* values were notably decreased. The enhancement in the lightness and redness and the reduction in yellowness of the fish fillets caused by LMH treatment could be attributed to factors such as protein oxidation and pigment degradation [27]. Remarkably, HIH derived from LMH showed the highest L* and a* values, while its b* values were significantly lower than those of the control group and were comparable to those of the LMH group. This could be a result of the suppression of high iron myoglobin conversion during the freezing process which, due to significant accumulation, leads to reduce a* values; whereas pigment degradation and the inherent color of the fish contribute to increased b* values [28]. Therefore, HIH showed potential as an active agent to preserve the coloration of frozen fish fillets.

### 3.3. Analyses of Moisture Loss

Moisture loss can lead to a loss of flavor and firmness in thawed meat, which can cause spoilage. The impacts of various treatments on thawing loss, cooking loss, and centrifugation loss of the fish fillets are shown in Table 2. Following three cycles of freezing and thawing, the control group exhibited significantly higher values (*p* < 0.05) for all three types of moisture loss compared with most of the treatment groups and fresh fish samples. This may be due to the growth of ice crystals as well as re-crystallization, which causes significant damage to muscle tissue and the structure of MFP [29]. In addition, damage to cell membranes leads to a loss of cytosol, promotes fat oxidation, and also leads to the oxidation and aggregation of proteins [30]. The three water loss rates (thawing, cooking, and centrifugation losses) of fish fillets treated with peptide solution immersion were significantly lower than those of the control group, in the order of LMH < TH < HMH < control, and the three water loss rates of LMH-treated fish fillets were reduced by 55.24%, 42.44%, and 44.94%, respectively, when compared with the control group. Furthermore, employment of the HIH treatment exhibited even more pronounced effects on water retention, with decreases in water loss of 55.48% (thawing), 48.49% (cooking), and 48.70% (centrifugation) relative to the control. These results suggest that HIH prevents the growth of ice crystals and the associated damage to cell membranes during the freeze-thaw process, thereby improving the water retention capacity of frozen fish fillets.

### 3.4. E-Nose Analysis

**Figure 1:**
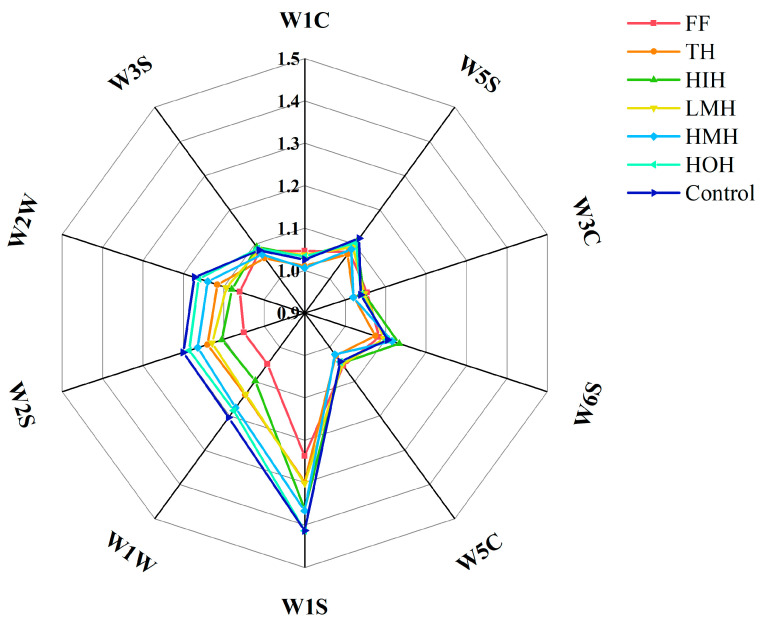
Radargrams of the response signals of the electronic nose of turbot after freeze-thaw cycles under different treatment conditions. Note: Different sensor response characteristics: W1S: methyl group; W1C: aromatic components (benzene); W5S: nitrogen oxides; W3C: aromatic components (ammonia); W6S: hydrogenide; W5C: short-chain alkanes, aromatic components; W1W: inorganic sulfides; W2S: alcohols; W2W: aromatic components (hydrocarbon), organosulfides; W3S: long-chain alkanes. FF, fresh fish; TH, tryptic hydrolysate solution; LMH, low-molecular-weight hydrolysate solution; HMH, high-molecular-weight hydrolysate solution; HIH, hydrophilic hydrolysate solution; HOH, hydrophobic hydrolysate solution; Control, aqueous solution.

The gas produced by fish after deterioration is mainly composed of volatile compounds; notably, aldehydes and ketones, unsaturated alcohols, and inorganic sulfides [31,32]. Figure 1 presents the results of the analysis of fish fillets under different treatments after freezing and thawing, where the control group exhibited the highest response values for sulfides (sensors W1W, W2W) and alcohols (sensor W2S), indicating a remarkable deterioration in the odor quality of the fish fillets. The reason for this may stem from the increased degree of protein denaturation and fat oxidation under repeated cycles of freezing and thawing, whereas alcohols are primarily generated through the reduction of sugars, amino acids, and aldehydes, as well as the enzymatic oxidation of fatty acids [33]. However, the response to these odor-inducing substances showed a gradual decrease across different treatments, with HMH, TH, and LMH exhibiting progressively lower sensor responses. Among these treatment groups, the HIH sample showed the most substantial reduction in response values for all three sensors, trailing only behind the fresh fish sample.

This result suggests that HIH reduced the production of undesirable odors during the freezing process, thereby maintaining the freshness and flavor of the fish.

### 3.5. Analysis of MFP Conformational Changes

#### 3.5.1. Fluorescence Spectroscopy

**Figure 2:**
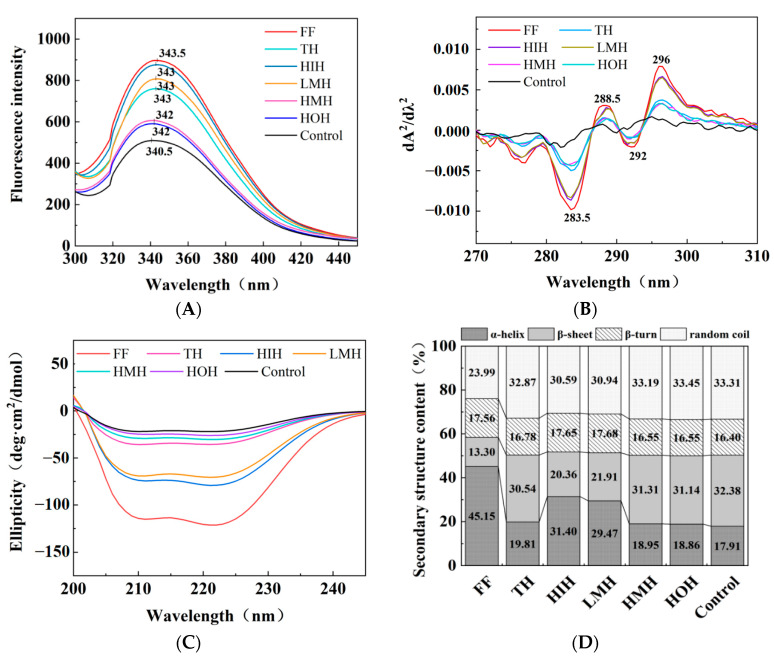
Effects of freeze-thaw cycles under different treatment conditions on MFP conformation in turbot. (**A**) Fluorescence spectrum; (**B**) UV second derivative spectra; (**C**) circular dichroism; and (**D**) secondary structure content. Note: FF, fresh fish; TH, tryptic hydrolysate solution; LMH, low-molecular-weight hydrolysate solution; HMH, high-molecular-weight hydrolysate solution; HIH, hydrophilic hydrolysate solution; HOH, hydrophobic hydrolysate solution; Control, aqueous solution.

Fluorescence spectroscopy was used to investigate the tertiary structure of proteins, focusing on protein oxidation and alterations in tryptophan residues. According to Figure 2A, they were all near 343 nm in the maximum fluorescence peak (λmax) across the four examined groups (i.e., HIH, LMH, HMH, and HOH). However, the λmax of the control MFP group exhibited a significant blueshift toward shorter wavelengths compared with the treatment groups. This shift indicates that the freeze-thaw process leads to greater aggregation of proteins and an increase in tryptophan residues in the polar microenvironment, thus exposing hydrophobic regions [34]. In contrast, the λmax of HIH, LMH, and TH samples was redshifted (shifted in the direction of larger wavelengths) relative to the control, HMH, and HOH, suggesting that the tryptophan microenvironment had become polarized due to unfolding of the protein structure [17].

Moreover, the fluorescence intensity (FI) showed a progressive decrease from HIH, LMH, TH, HMH to HOH. This trend could be attributed to the disruption of MFP structure due to ice crystal formation during freeze-thaw cycles, leading to protein aggregation and encapsulation of tryptophan within hydrophobic domains, resulting in reduced FI. The HIH sample showed the highest FI among the treatment groups, second only to the fresh fish (FF) group, indicating its superior ability to preserve the tertiary structure of MFP, thus minimizing protein cross-linking and aggregation throughout the freeze-thaw process [22].

#### 3.5.2. UV Absorption Spectroscopy

Changes in the tertiary structure of proteins can be inferred from alterations in the microenvironments surrounding specific amino acid residues such as tryptophan (Trp) and tyrosine (Tyr) [20]. Figure 2B shows the UV second-order derivative spectra of MFP, where absorption peaks at 288.5 nm were attributed to both Trp and Tyr residues, and a distinct peak at 296 nm was associated with Trp [13,15]. The absorption peaks at 296 nm of the control group were blueshifted when compared with those of the other groups, suggesting that protein oxidation occurred during freezing and thawing, resulting in the exposure and aggregation of hydrophobic residues. Interestingly, HIH and LMH samples were redshifted (i.e., shifted to longer wavelengths) at the 296 nm peak relative to the TH, HMH, HOH, and control groups, suggesting a stabilization effect of HIH and LMH treatments on the protein structure against oxidation.

Additionally, the TH, HMH, and HOH samples showed lower absorption at this wavelength compared to those of the HIH-treated group. This implies that HIH treatment is particularly effective in reducing protein oxidation and aggregation, thereby preserving the tertiary structure of proteins more effectively than other treatments. This finding is consistent with the fluorescence spectroscopy results.

#### 3.5.3. CD

The secondary conformational changes of proteins were determined through CD spectroscopy [35]. CD spectral images and secondary structure content maps of MFP under different freeze-thaw cycle treatments are shown in Figure 2C,D. As shown in Figure 2C, there were two notable negative CD peaks (210 nm, 222 nm) in the FF group, which were attributed to the presence of an α-helical structure [36]. The intensity of these peaks gradually decreased with the addition of HIH and LMH, while there were no significant changes compared with the TH, HMH, HOH, and control groups. As shown in Figure 2D, the FF group showed the highest α-helix content, and the content of α-helices gradually decreased from the HIH, LMH, TH, HMH, HOH to the control group, with the highest content of β-folded and irregularly curled content observed in the control group. This indicates that the main secondary structure in the natural MFP was the α-helical structure, which was partly converted into β-folded and irregularly curled during the process of deconvolution of the curly structure. This was due to ice crystal growth and recrystallization, which leads to the exposure of internal hydrophobic groups and protein aggregation, resulting in elevated levels of β-folding and irregular curling [37]. The results showed that HIH presented the ability to inhibit the growth and re-crystallization of ice crystals, thus reducing the degree of protein aggregation during the freeze-thaw process and improving the conformational stability of proteins. These results were consistent with those of the moisture loss assay.

### 3.6. Analysis of Microstructure

**Figure 3:**
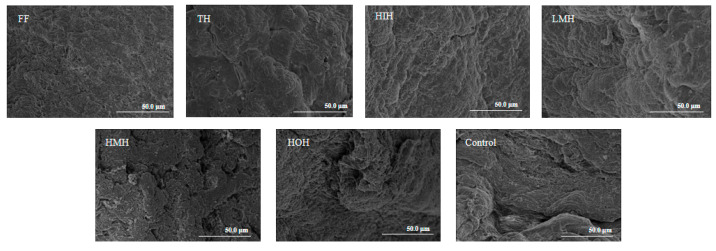
Effects of freeze-thaw cycles under different treatment conditions on the microstructure of turbot. Note: FF, fresh fish; TH, tryptic hydrolysate solution; LMH, low-molecular-weight hydrolysate solution; HMH, high-molecular-weight hydrolysate solution; HIH, hydrophilic hydrolysate solution; HOH, hydrophobic hydrolysate solution; Control, aqueous solution.

The microstructures of the fish fillet surfaces after the freeze-thaw treatments are shown in Figure 3. The surface of the FF group samples showed a dense and smooth fibrous reticulation structure, in contrast to the control group, which displayed significant structural degradation characterized by pronounced fissures and extensive fibrous gaps. Both the HMH and TH samples presented varying content of fibrous discontinuities, which may be attributed to the ice crystals in the freezing and thawing process damaging the fibrous structure, leading to dehydration of the muscle cells [37]. In contrast, the LMH group demonstrated more defined fibrous tissues with reduced inter-fiber spacing. Moreover, the surface fiber texture of HIH fish fillets after further separation was notably refined and compact. These results indicate that HIH promoted better water retention, and this phenomenon was supported by the moisture loss results (Table 2). In summary, the treatment of fish fillets with HIH reduced tissue damage, protected the fiber mesh structure, and improved the stability of fish meat after the freeze-thaw cycles.

### 3.7. Analysis of MFP Oxidative Denaturation

#### 3.7.1. Particle Size and Zeta Potential

**Figure 4:**
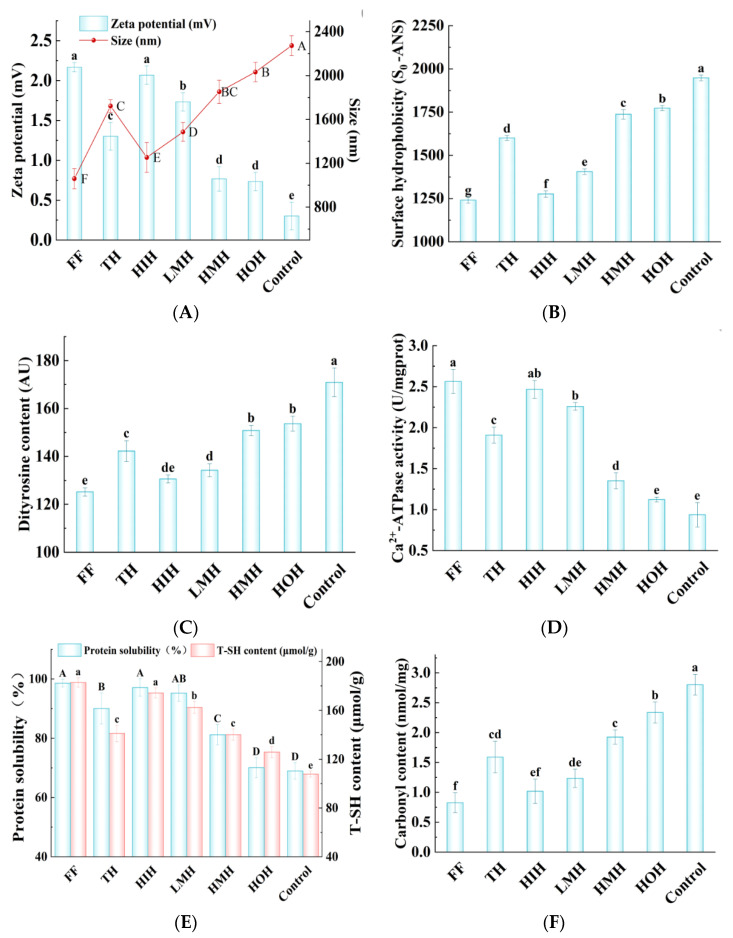
Effects of freeze-thaw cycles under different treatment conditions on the degree of oxidation and aggregation of MFP in turbot. (**A**) Zeta potential and particle size; (**B**) surface hydrophobicity; (**C**) dityrosine content; (**D**) protein solubility and total sulfhydryl content; (**E**) Ca^2+^-ATPase activity; and (**F**) carbonyl content. Note: FF, fresh fish; TH, tryptic hydrolysate solution; LMH, low-molecular-weight hydrolysate solution; HMH, high-molecular-weight hydrolysate solution; HIH, hydrophilic hydrolysate solution; HOH, hydrophobic hydrolysate solution; Control, aqueous solution. Note: In the significance analysis, the letters from A to F and a to f represent the values from large to small, and different letters represent significant differences (*p* < 0.05).

Zeta potential (ζ) is an indicative index of colloidal stability in protein systems, indicated by the charge of proteins. Furthermore, the effective particle size reflects the extent of protein aggregation and oxidation [38,39]. When the ζ absolute value (|ζ|) of the protein solution is smaller, there are fewer charges on the surface of proteins, which could decrease electrostatic repulsion and cause the system of protein molecules to tend to aggregate, thus increasing the effective particle size; therefore, the stability of the solution will be reduced [17,40]. As shown in Figure 4A, the average ζ values of the control, HMH, TH, LMH, and FF group samples were 0.30, 0.76, 1.30, 1.73, and 2.16 mV, respectively. These values were inversely correlated with the effective particle sizes, measured at 2273.40, 1851.96, 1722.00, 1485.60, and 1059.20 nm, indicating a trend where increasing zeta potential corresponded to decreasing particle size. The reduction in particle size may be attributed to the inhibition of ice crystal formation by the treatments, thus reducing protein denaturation and aggregation. Notably, the HIH sample showed a ζ of 2.06 mV and an effective particle size of 1253.33 nm, ranking it second to the FF group in terms of performance. This indicates that the HIH treatment could effectively inhibit the aggregation of proteins during the freeze-thaw cycles, thereby enhancing the stability of the protein system.

#### 3.7.2. So-ANS

Surface hydrophobicity serves as a critical parameter for assessing alterations in the physicochemical properties of MFP, specifically in assessing protein denaturation levels [41]. The measurement of surface hydrophobicity was performed using the 1-anilino-8-naphthalene sulfonate (ANS) binding assay, with the So-ANS value representing the degree of hydrophobicity. As shown in Figure 4B, the control group showed the highest So-ANS value (1947.60), which can be attributed to the oxidative processes leading to the unfolding of protein structures and the exposure of hydrophobic residues that were previously buried within the protein core [42]. In contrast, the HMH, TH, and LMH treatments resulted in a progressive decline in So-ANS values relative to the control group as well as a gradual transformation of the β-folding of MFP into an α-helical structure (Figure 2D) phenomena indicating that these processing methods may mitigate protein denaturation and make the protein structure more stable. It is worth mentioning that the So-ANS value under HIH was significantly lower than those of all other treatment groups (*p* < 0.05). These results indicated that the HIH treatment had a pronounced protective effect against the denaturation impact of freeze-thaw cycles on the protein structure in fish fillets through reducing the exposure of hydrophobic domains.

#### 3.7.3. Dityrosine Content

The process of oxidation in MFP may lead to the covalent cross-linking of tyrosine residues with other amino acids, including the formation of dityrosine through the complexation of two tyrosine molecules. Therefore, quantification of dityrosine levels serves as an indicator of oxidative stress within MFP [43]. The dityrosine content in different samples after freeze-thawing is shown in Figure 4C. Compared with the control group, HMH, TH, and LMH treatments resulted in decreases in dityrosine content by 20.10, 28.70, and 36.70 Absorbance Units (AU), respectively. Notably, the LMH-treated samples exhibited a significantly lower dityrosine level than those treated with HMH and TH, indicating a more pronounced reduction in oxidative cross-linking within this group (*p* < 0.05).

Furthermore, the HIH treatment led to an even more substantial decrease in dityrosine content, amounting to a reduction of 40.37 AU compared to the control. The difference in dityrosine levels between HIH-treated samples and those treated with LMH was not statistically significant, suggesting that both treatments are comparably effective in mitigating oxidative stress. These results indicate the potential of HIH and LMH treatments in preserving the integrity and functionality of muscle proteins through inhibiting the degree of oxidative damage under freeze-thawing conditions.

#### 3.7.4. Ca^2+^-ATPase Activity

Ca^2+^-ATPase activity is closely related to the myosin head structure, reflecting MFP structural integrity, and diminishes when the sulfhydryl content of the myosin head is oxidized [44]. As shown in Figure 4D, the control MFP exhibited the lowest Ca^2+^-ATPase activity and the most significant decline, stemming from the formation of ice crystals within the myosin head region. These ice crystals facilitate the oxidation of sulfhydryl groups into disulfide bonds, ultimately resulting in oxidative denaturation of the protein. The Ca^2+^-ATPase activity in LMH, TH, and HMH samples was increased by 58.41%, 50.79%, and 30.37%, respectively, relative to the control group, suggesting a better protective effect of LMH on Ca^2+^-ATPase activity. HIH was further isolated from LMH to significantly increase Ca^2+^-ATPase activity (which increased by 61.94%). HIH had the highest Ca^2+^-ATPase activity, suggesting that it could inhibit the sulfhydryl oxidation of MFP during freeze-thawing, which resulted in a lower degree of Ca^2+^-ATPase activity destruction and maintenance of the intact myosin head structure.

#### 3.7.5. Protein Solubility

Decreased protein solubility leads to protein denaturation and reduced muscle quality [45]. As shown in Figure 4E, the control group showed the lowest solubility (at 68.96%), likely due to structural damage from ice crystals during the freeze-thaw cycles, wherein the dissociation of bound water prompts protein denaturation via freezing [46]. Additionally, hydrophobic residues facilitate protein aggregation and solubility reduction due to hydrophobic interactions [47]. The solubility of LMH, TH, and HMH samples increased by 38.12%, 30.61%, and 17.72%, respectively, compared to the control. The solubility of HIH and HOH samples, obtained after separation from LMH again, was increased by 40.88% and 1.60%, respectively, compared to the control group, with HIH achieving the highest solubility improvement. These results suggest that HIH successfully blocked the exposure of reactive sulfhydryl groups and hydrophobic residues, thereby preventing the development of disulfide bonds (Figure 4E) and surface hydrophobicity (Figure 4B). Additionally, it effectively mitigated the mechanical damage caused by ice crystals on proteins and inhibited the oxidative denaturation of MFP.

#### 3.7.6. Total Sulfhydryl Content

Sulfhydryl groups in MFP are prone to oxidation, leading to disulfide bonds and protein cross-linking, making their content a key marker of protein oxidation [48]. The changes in sulfhydryl content of different MFP samples after freeze-thawing is shown in Figure 4E. Among all the groups, the control group exhibited the lowest sulfhydryl content, which could be attributed to protein oxidation during freezing resulting in the formation of sulfhydryl products, or alterations in the spatial structure of proteins that expose sulfhydryl groups, which subsequently oxidize and manifest as disulfide bonds [49]. Relative to the control group, the total sulfhydryl content of LMH, TH, and HMH samples was increased by 54.58, 33.28, and 32.31 μmol/g, respectively. Notably, the total sulfhydryl content of further isolated HIH (174.28 μmol/g) was significantly higher than that of the other sample groups. This result indicates that HIH could decrease MFP oxidation during freeze-thawing; this change in sulfhydryl content is consistent with the CD and Ca^2+^-ATPase activity results.

#### 3.7.7. Carbonyl Content

Protein carbonylation occurs when an oxygen radical attacks the amino group in the side chain of an amino acid residue, leading to the formation of a carbonyl group [50]. Carbonyl content is one of the most commonly used indicators for evaluating protein oxidation [51]. As shown in Figure 4F, the control group showed the highest degree of carbonylation, reaching 2.80 nmol/mg; the development of ice crystals often results in cell rupture, subsequently releasing oxidative enzymes and pro-oxidants that hasten protein oxidation and promote the formation of carbonyls [37]. In contrast, treatment with LMH, TH, and HMH decreased the carbonyl level by 56.07%, 43.21%, and 31.07%, respectively, relative to the control, highlighting LMH’s superior efficacy in reducing carbonylation. One plausible explanation is the suppression of ice crystal formation during the freezing process, minimizing cellular damage and thereby preventing the oxidation of MFP. The HIH phase after further isolation of LMH showed a carbonyl level reduction of 63.57% compared to control group. Thus, it significantly inhibited carbonylation, with the lowest carbonyl content compared to the other groups. This result suggests that HIH significantly lowers protein oxidation, impedes the formation of ice crystals, and lowers the carbonyl content.

## 4. Conclusions

The obtained results demonstrated that the cryoprotective properties of the HIH fraction (further isolated from LMH) on fish meat were associated with the alleviation of ice crystal damage and the inhibition of protein oxidation. The texture property, moisture loss, E-nose, and SEM results showed that HIH pre-treatment successfully improved water retention in the fish samples and reduced the decrease in quality caused by ice crystals. The fluorescence spectroscopy, UV absorption spectroscopy, CD spectroscopy, So-ANS, particle size, and zeta potential results indicated that HIH pre-treatment prevented the exposure of hydrophobic groups, thus effectively inhibiting protein aggregation and cross-linking, as well as stabilizing the protein structure. The results of color, dityrosine, Ca^2+^-ATPase activity, solubility, sulfhydryl content, and carbonyl content assessments indicated that HIH pre-treatment inhibited the oxidative denaturation of MFP. In summary, the HIH component had the most effective cryoprotective effect of TH from croceine croaker after freeze-thaw treatment, which provides insights for the further study of antifreeze peptides for marine fish and offers valuable insights regarding their potential application in the food industry.

## Figures and Tables

**Table 1:** Effects of freeze-thaw cycles under different treatments on the textural properties of turbot. Note: FF, fresh fish; TH, tryptic hydrolysate solution; LMH, low-molecular-weight hydrolysate solution; HMH, high-molecular-weight hydrolysate solution; HIH, hydrophilic hydrolysate solution; HOH, hydrophobic hydrolysate solution; Control, aqueous solution.

Samples	Hardness (g)	Elastic (mm)	Cohesion (g.s)	Adhesion (g)	Chewiness (mJ)	Reversibility (mm)
FF	65.60 ± 6.25 ^a^	1.00 ± 0.08 ^a^	3.32 ± 0.06 ^a^	18.73 ± 3.66 ^b^	18.43 ± 4.03 ^c^	0.41 ± 0.01 ^a^
TH	44.65 ± 5.43 ^c^	0.96 ± 0.06 ^ab^	3.24 ± 0.05 ^b^	24.41 ± 4.38 ^b^	22.98 ± 5.20 ^bc^	0.38 ± 0.04 ^ab^
LMH	46.17 ± 5.47 ^c^	0.97 ± 0.05 ^ab^	3.25 ± 0.05 ^b^	22.77 ± 4.41 ^b^	21.33 ± 5.24 ^bc^	0.39 ± 0.03 ^ab^
HIH	57.62 ± 4.98 ^b^	0.98 ± 0.04 ^ab^	3.26 ± 0.02 ^b^	20.61 ± 3.05 ^b^	19.42 ± 3.29 ^bc^	0.40 ± 0.02 ^a^
HMH	40.78 ± 5.34 ^cd^	0.95 ± 0.05 ^ab^	3.23 ± 0.02 ^b^	26.58 ± 4.29 ^a^	23.08 ± 3.18 ^ab^	0.37 ± 0.03 ^ab^
HOH	39.30 ± 5.30 ^cd^	0.93 ± 0.06 ^ab^	3.21 ± 0.02 ^b^	27.19 ± 4.26 ^a^	24.72 ± 3.16 ^ab^	0.37 ± 0.03 ^ab^
Control	36.95 ± 6.26 ^d^	0.92 ± 0.07 ^b^	3.18 ± 0.05 ^c^	28.64 ± 4.63 ^a^	25.87 ± 4.53 ^a^	0.37 ± 0.02 ^b^

Note: In the significance analysis, the letters from a to d represent the values from large to small, and different letters represent significant differences (*p* < 0.05).

**Table 2:** Effects of freeze-thaw cycles under different treatment conditions on color difference and moisture loss in turbot samples. Note: FF, fresh fish; TH, tryptic hydrolysate solution; LMH, low-molecular-weight hydrolysate solution; HMH, high-molecular-weight hydrolysate solution; HIH, hydrophilic hydrolysate solution; HOH, hydrophobic hydrolysate solution; Control, aqueous solution.

Samples	L*	a*	b*	Thawing Loss (%)	Cooking Loss (%)	Centrifugal Loss (%)
FF	56.07 ± 0.51 ^a^	2.78 ± 0.10 ^a^	1.49 ± 0.12 ^e^	--	5.65 ± 0.65 ^d^	2.35 ± 0.32 ^e^
TH	47.65 ± 5.43 ^c^	0.96 ± 0.06 ^ab^	3.24 ± 0.05 ^b^	7.30 ± 0.55 ^d^	9.78 ± 0.45 ^b^	5.29 ± 0.85 ^c^
LMH	52.28 ± 0.91 ^b^	1.74 ± 0.05 ^b^	2.42 ± 0.13 ^d^	5.64 ± 0.49 ^e^	7.61 ± 1.82 ^c^	4.24 ± 0.09 ^cd^
HIH	54.71 ± 0.92 ^a^	2.06 ± 0.05 ^b^	2.08 ± 0.09 ^d^	5.61 ± 0.40 ^e^	6.81 ± 0.13 ^cd^	3.95 ± 0.23 ^d^
HMH	46.51 ± 0.97 ^d^	1.47 ± 0.14 ^c^	3.25 ± 0.05 ^b^	9.10 ± 0.60 ^c^	11.11 ± 0.25 ^b^	6.44 ± 0.34 ^b^
HOH	44.88 ± 0.98 ^c^	1.38 ± 0.14 ^d^	3.18 ± 0.05 ^b^	11.24 ± 0.52 ^b^	11.12 ± 0.60 ^b^	6.85 ± 0.76 ^a^
Control	42.38 ± 0.80 ^e^	1.13 ± 0.06 ^d^	3.52 ± 0.18 ^a^	12.60 ± 0.86 ^a^	13.22 ± 0.22 ^a^	7.70 ± 0.63 ^a^

Note: In the significance analysis, the letters from a to e represent the values from large to small, and different letters represent significant differences (*p* < 0.05). -- indicates that this indicator does not need to be measured for this experimental group.

## Data Availability

The original contributions presented in the study are included in the article, further inquiries can be directed to the corresponding authors.
